# Prospective Longitudinal Assessment of Central Hemodynamic Profile in Normotensive, Hypertensive and Preeclamptic Women in Pregnancy and Postpartum

**DOI:** 10.3390/jcm15218247

**Published:** 2026-10-23

**Authors:** Ludwina Szczepaniak-Chicheł, Dawid Lipski, Paweł Uruski, Andrzej Tykarski

**Affiliations:** Department of Hypertensiology, Angiology and Internal Medicine, Poznan University of Medical Sciences, Długa 1/2, 61-848 Poznan, Poland; dlipski@ump.edu.pl (D.L.); puruski@ump.edu.pl (P.U.); tykarski@ump.edu.pl (A.T.)

**Keywords:** augmentation index, carotid–femoral pulse wave velocity, central aortic blood pressure, preeclampsia, pregnancy hypertension, pulse pressure amplification, pulse wave analysis, prospective longitudinal study

## Abstract

**Background/Objectives**: Central hemodynamic assessment may provide information complementary to brachial blood pressure in hypertensive disorders of pregnancy, but its clinical predictive value remains uncertain. This prospective longitudinal study evaluated central aortic systolic blood pressure (AoSBP), augmentation index (AIx), augmentation index normalized to 75 beats/min (AIxHR75), pulse pressure amplification (PPAmpl), and carotid–femoral pulse wave velocity (cfPWV) in pregnant women followed at a tertiary outpatient clinic in Poznan, Poland. **Methods**: Between 2017 and 2023, 241 consecutive pregnant women were examined during pregnancy and postpartum using applanation tonometry (SphygmoCor^®^ PVx). The cohort included 40 normotensive controls, 152 women with hypertension without preeclampsia (HT), and 49 women with hypertension complicated by preeclampsia (HT + PE). Longitudinal profiles were analyzed using prespecified gestational periods, and additional mixed-effects and exploratory ROC analyses were performed in the subgroup with complete observations from 19–22 to 31–34 weeks of gestation. **Results**: AoSBP was consistently lower in controls than in hypertensive women despite pharmacological control of brachial blood pressure in the hypertensive groups. In the complete-case analysis, AoSBP differentiated the groups most clearly: it was higher in HT than in controls from 19–22 to 31–34 weeks and increased further in HT + PE from 23–26 weeks onward. PPAmpl showed no significant group, period, or interaction effect, whereas AIx and AIxHR75 changed over time but did not show robust between-group differences after Holm correction. Exploratory ROC analyses showed only moderate discrimination for selected parameters and should be considered hypothesis-generating. **Conclusions**: Women who developed preeclampsia showed an altered central hemodynamic phenotype compared with normotensive pregnancies and uncomplicated hypertensive pregnancies. These findings indicate association and discrimination at the group level, not clinically validated early prediction. External validation and multivariable models including conventional clinical variables and blood pressure measures are required before clinical implementation.

## 1. Introduction

Hypertensive disorders of pregnancy, including chronic hypertension, gestational hypertension, and preeclampsia, remain one of the leading causes of maternal and fetal morbidity and mortality worldwide. Hypertensive disorders of pregnancy affect approximately 5–10% of pregnancies and represent a significant clinical, diagnostic, and prognostic challenge in modern obstetrics [1,2]. Preeclampsia is of particular importance, as it is a multisystem disorder associated with endothelial dysfunction, abnormal placental perfusion, and an increased risk of cardiovascular complications both during pregnancy and later in life [3,4].

In recent years, central hemodynamic assessment has attracted interest as a complementary approach to conventional brachial blood pressure measurement. These indices represent related but distinct physiological concepts. Central aortic blood pressure reflects the pressure load imposed on the proximal arterial tree and left ventricle; AIx reflects the magnitude and timing of wave reflection and is influenced by heart rate, body size, vascular tone, and arterial properties; PPAmpl describes the relationship between peripheral and central pulse pressure; and cfPWV is generally considered the most direct non-invasive index of aortic stiffness [5,6,7]. This distinction is important because these measurements should not be grouped uncritically as equivalent arterial stiffness parameters.

Physiological pregnancy is associated with major hemodynamic changes, including increased blood volume and cardiac output, reduced systemic vascular resistance, altered vascular tone, and changes in arterial compliance. These adaptations are reflected by lower central pressure and reduced wave reflection during mid-pregnancy, followed by a partial return toward pre-pregnancy vascular tone near term [8]. In hypertensive disorders of pregnancy, these adaptive changes may be attenuated, and several studies have reported higher central pressure, altered wave reflection, and increased arterial stiffness indices in women with gestational hypertension or preeclampsia [9,10].

However, the role of central hemodynamic measurements in pregnancy remains unsettled. Adult cardiovascular studies support associations between central pressure, arterial stiffness, and adverse cardiovascular outcomes, but this does not directly establish superior diagnostic or predictive performance in pregnancy [5,6,7]. A clear distinction is therefore needed between pathophysiological association, group-level discrimination, and clinically useful prediction. The latter requires evidence that a marker improves individual risk classification beyond established clinical variables, conventional blood pressure, Doppler findings, or biochemical markers.

Previous studies have suggested that women with preeclampsia may show higher central systolic pressure, increased arterial stiffness, altered wave reflection, and reduced pressure amplification compared with normotensive pregnancy or hypertension without preeclampsia [9,10,11]. Nevertheless, most available data derive from cross-sectional assessments or from specific gestational windows, commonly 11–13 or 20–24 weeks. Longitudinal studies simultaneously assessing central pressure, wave reflection, PPAmpl, and cfPWV across pregnancy and postpartum remain limited.

The aim of the present study was to evaluate longitudinal profiles of AoSBP, AIx, AIxHR75, PPAmpl, and cfPWV in normotensive pregnancies, uncomplicated hypertensive pregnancies, and hypertensive pregnancies complicated by preeclampsia. In this manuscript, “uncomplicated hypertension” refers to chronic or gestational hypertension without clinical or laboratory features of preeclampsia. These entities were analyzed together because the primary objective was to compare hypertensive pregnancies with and without preeclampsia; subgroup analyses by chronic versus gestational hypertension were not sufficiently powered. We hypothesized that women who developed preeclampsia would show a distinct central hemodynamic phenotype compared with women with uncomplicated hypertension and normotensive controls.

## 2. Materials and Methods

### 2.1. Study Design and Participants

This prospective longitudinal observational study was conducted in a cohort of pregnant women followed at a tertiary outpatient clinic between May 2017 and October 2023. The study population consisted of 241 pregnant women who agreed to participate and were monitored throughout pregnancy and the postpartum period. Women in hypertensive groups were included consecutively, while normotensive controls were invited during routine gynecological visits.

Eligibility criteria were defined separately for the hypertensive cohort and the normotensive control group. Women with chronic hypertension were eligible if they had a diagnosis of chronic arterial hypertension before pregnancy or before 20 weeks of gestation and were receiving antihypertensive treatment, irrespective of the type or number of antihypertensive agents used. Women with pregnancy-induced (gestational) hypertension were eligible if hypertension was diagnosed after 20 weeks of gestation, with blood pressure >140/90 mmHg, no diagnosis of hypertension before pregnancy, and ongoing antihypertensive treatment, irrespective of the treatment regimen.

Normotensive pregnant controls were eligible if they had office blood pressure <140/90 mmHg, no clinically significant heart, kidney, or connective tissue disease, no antihypertensive therapy, and provided written informed consent.

Exclusion criteria included age <18 years, previously diagnosed schizophrenia or bipolar disorder, and a diagnosis of heart failure. At the cohort level, multiple pregnancy, kidney disease, and pre-existing diabetes were not exclusion criteria. No participant had a diagnosed connective tissue disease.

Gestational age at written informed consent was 15.0 (11.4; 16.8) weeks in the whole cohort, 15.5 (13.3; 16.9) weeks in controls, 15.0 (11.2; 16.4) weeks in the HT group, and 12.3 (10.3; 19.3) weeks in the HT + PE group. Methyldopa was the most frequently used antihypertensive agent at enrollment: 35 women (56.5%) in the subgroup with medication data, 28 (66.7%) in the HT group, and 6 (75.0%) in the HT + PE group. Calcium-channel blockers were used in 5 women (8.1%) overall, including 4 (9.5%) in the HT group and 1 (12.5%) in the HT + PE group. Metoprolol was used by 8 women (12.9%) overall, including 4 (9.5%) in the HT group and 4 (50.0%) in the HT + PE group. One woman in the HT + PE group received a diuretic (12.5% of HT + PE; 1.6% overall).

After enrollment, participants were scheduled for regular follow-up visits approximately every four weeks from the first visit until delivery. The visit schedule remained flexible and could be modified depending on clinical status and blood pressure control. Postpartum follow-up visits were conducted at 6–8 weeks, 6 months, and 12 months after delivery.

In total, 1232 clinical visits were performed and included in the analysis. No formal sample-size calculation was performed; the sample size was determined by feasible consecutive recruitment within a real-life longitudinal registry. The study was initiated and conducted independently by the investigators, and no financial support or incentives were provided by device manufacturers.

This study was reported in accordance with the STROBE statement checklist for cohort studies. The completed STROBE checklist for the presented study is available in the Supplementary Materials (Supplementary Table S1).

### 2.2. Clinical Assessment

At each visit, data on clinical status and pregnancy complications were collected on the basis of ultrasound findings, laboratory tests, and clinical assessment. Office blood pressure was measured and arterial stiffness and pulse wave parameters were assessed by applanation tonometry. Additional variables collected during follow-up included parity, smoking status, comorbidities, acetylsalicylic acid use, body mass, gestational age at delivery, mode of delivery, and neonatal outcomes. These variables were included in the descriptive analysis.

Antihypertensive treatment in patients with hypertension was managed individually based on blood pressure values (office and 24 h ABPM one each visit) and clinical condition, in accordance with current guideline recommendations [12,13]. Treatment was adjusted during follow-up visits to achieve safe and adequate blood pressure control throughout pregnancy.

For statistical analysis, participants were classified into three groups: normotensive pregnancy (control group), hypertension without preeclampsia (HT group), and hypertension complicated by preeclampsia (HT + PE group). The HT group included both chronic hypertension and gestational hypertension, provided that no maternal, fetal, laboratory, or placental criteria of preeclampsia were present. The two forms of uncomplicated hypertension were intentionally combined because the study focused on the hemodynamic differences associated with preeclampsia within hypertensive pregnancy; the number of complete longitudinal observations was insufficient for adequately powered subgroup analyses by hypertension type.

### 2.3. Office Blood Pressure Measurements

Office blood pressure was measured using a validated oscillometric device (OMRON^®^ 705IT; Omron Healthcare Co., Ltd., Kyoto, Japan). Office blood pressure values were recorded as the average of three consecutive measurements taken at least 60 s apart, between 8:00 and 11:00 a.m., in a quiet, temperature-controlled environment, in the presence of a physician, in accordance with European Society of Hypertension and European Society of Cardiology guidelines [12,13].

### 2.4. Pulse Wave Analysis and Pulse Wave Velocity Measurements

Central hemodynamic parameters were assessed non-invasively using applanation tonometry (SphygmoCor^®^, AtCor Medical, Sydney, Australia), a validated method for pulse wave analysis and pulse wave velocity measurement. All measurements were performed in a quiet, temperature-controlled room after at least 10 min of rest in the supine position. Radial artery pressure waveforms were recorded using a high-fidelity micromanometer, and a validated generalized transfer function was applied to derive the corresponding central aortic pressure waveform. AoSBP, AIx, AIxHR75, and PPAmpl were calculated automatically by the device software. Because AIx is heart-rate dependent, AIxHR75 was used to facilitate comparison between participants and visits. PPAmpl was defined as the ratio of peripheral to central pulse pressure and was interpreted as an index of central-to-peripheral pressure amplification [14,15].

Carotid–femoral pulse wave velocity (PWV) was measured as an index of aortic stiffness using sequential recordings of carotid and femoral artery waveforms. The distance between measurement sites was measured over the body surface using a standardized protocol. Pulse wave velocity was calculated as the distance divided by the transit time between the carotid and femoral pulse waveforms [14,15].

All pulse wave analysis and pulse wave velocity measurements were performed by a single trained investigator to minimize interobserver variability. Measurements were repeated if signal quality was insufficient according to device quality control criteria.

### 2.5. Definitions

Arterial hypertension in the study was defined based on ambulatory 24 h blood pressure monitoring results, with mean daytime systolic/diastolic blood pressure ≥135/85 mmHg and/or nighttime blood pressure ≥120/75 mmHg or treatment with antihypertensive drugs [12,13]. ABPM was performed with a validated device (AND TM-2430; A&D Company, Limited; Tokyo, Japan), with measurements done every 15 min during the day (7 AM to 10 PM) and every 30 min at night. Gestational hypertension was defined as hypertension occurring after 20 weeks of gestation in previously normotensive women, with normalization of blood pressure within 42 days postpartum. Chronic hypertension was defined as hypertension present before pregnancy or diagnosed before 20 weeks of gestation and persisting beyond 42 days postpartum. Preeclampsia was defined according to current ESC/ESH guidelines as hypertension accompanied by one or more of the following new-onset conditions occurring after 20 weeks of gestation: proteinuria, acute kidney injury, liver involvement, neurological complications, hematological complications, or uteroplacental dysfunction [16].

### 2.6. Statistical Analysis

Longitudinal measurements were aggregated into prespecified study periods by averaging values from adjacent visits within each patient whenever at least one measurement was available in a given time window. For each aggregated period, central tendency was described using medians with first and third quartiles, and parameter profiles were constructed separately for the control, HT, and HT + PE groups. The prespecified pairwise comparisons were control versus HT and HT versus HT + PE. Control versus HT + PE comparisons were not used as the main inferential framework because the clinically relevant question was whether women with uncomplicated hypertension differed from those with hypertension complicated by preeclampsia. Between-group differences at each time point were assessed using the Mann–Whitney U test. A two-sided *p* value <0.05 was considered statistically significant.

Additional longitudinal analyses were performed in the subgroup with both PWA and cfPWV assessments available at M5, M6, M7, and M8. For each parameter, a linear mixed-effects model was fitted by restricted maximum likelihood, with study group, period, and the group × period interaction as fixed effects and a patient-specific random intercept. Model-derived estimated marginal means (EMMs) and 95% confidence intervals were calculated for each group and period. Within-period contrasts were evaluated using Holm adjustment for multiple comparisons.

Exploratory ROC analyses were restricted to the complete M5–M8 cohort. Because the cutoffs were selected using the Youden index and derived and evaluated in the same cohort, these analyses were considered hypothesis-generating and were not interpreted as externally validated prediction models. The area under the curve (AUC), cutoff value, sensitivity, and specificity were reported where available. Incremental predictive value beyond conventional clinical variables, office blood pressure, ambulatory blood pressure monitoring, Doppler indices, or biochemical markers was not assessed. Statistical analyses were performed with Statistica (version 13.3, TIBCO Software Inc., Palo Alto, CA, USA) and Python (version 3.12.10; Python Software Foundation, Wilmington, DE, USA) using NumPy (version 2.3.5), pandas (version 3.0.6), SciPy (version 1.17.1), and statsmodels (version 0.15.0). Figures were prepared using Matplotlib (version 3.10.7; NumFOCUS, Austin, TX, USA).

### 2.7. Ethical Approval

The study protocol was approved by the Regional Institutional Medical Research Ethics Committee (approval no. 141/07, dated 1 February 2007; continuation and extension approval no. 1171/19, dated 5 December 2019) and was conducted in accordance with the Declaration of Helsinki and its later amendments [17]. All participants provided written informed consent before inclusion and were informed that they could withdraw at any stage without consequences.

## 3. Results

The study included 40 normotensive pregnant women and 201 women with arterial hypertension. Among hypertensive women, 145 had pre-existing chronic hypertension and 56 had gestational hypertension. Preeclampsia developed in 49 hypertensive women (HT + PE), including 27 women with chronic hypertension and 22 women with gestational hypertension. The remaining 152 hypertensive women constituted the HT group, including 118 women with chronic hypertension and 34 women with gestational hypertension. Preeclampsia was diagnosed at a mean gestational age of 32.8 weeks (range, 20–39 weeks). Baseline and pregnancy characteristics are presented in Table 1, and participation at each study visit is shown in Table 2.

Women in the HT + PE group delivered earlier and had lower neonatal birth weight and lower 1-min Apgar scores than women in the HT group or controls (*p* < 0.05). Preeclampsia was also associated with a higher frequency of caesarean delivery, fetal growth restriction (FGR), and lower use of acetylsalicylic acid prophylaxis. No significant differences were observed between controls and women with uncomplicated hypertension for these obstetric and neonatal outcomes. Age, parity, pre-pregnancy BMI, obesity prevalence, pregnancy weight gain, gestational diabetes, and smoking status did not differ significantly between the analyzed groups.

Eight adverse pregnancy or neonatal events occurred: three miscarriages not related to elevated blood pressure, two intrauterine fetal deaths (one at 23 weeks of gestation associated with severe early preeclampsia and one associated with Edwards syndrome), and three neonatal deaths after preterm delivery not attributed to elevated blood pressure or antihypertensive treatment.

Peripheral systolic and diastolic blood pressure (pSBP and pDBP), heart rate (HR), central aortic pressure, pulse wave analysis parameters, and cfPWV across pregnancy and postpartum are summarized in Table 3, Table 4 and Table 5 for the control, HT, and HT + PE groups, respectively.

**Figure 1:**
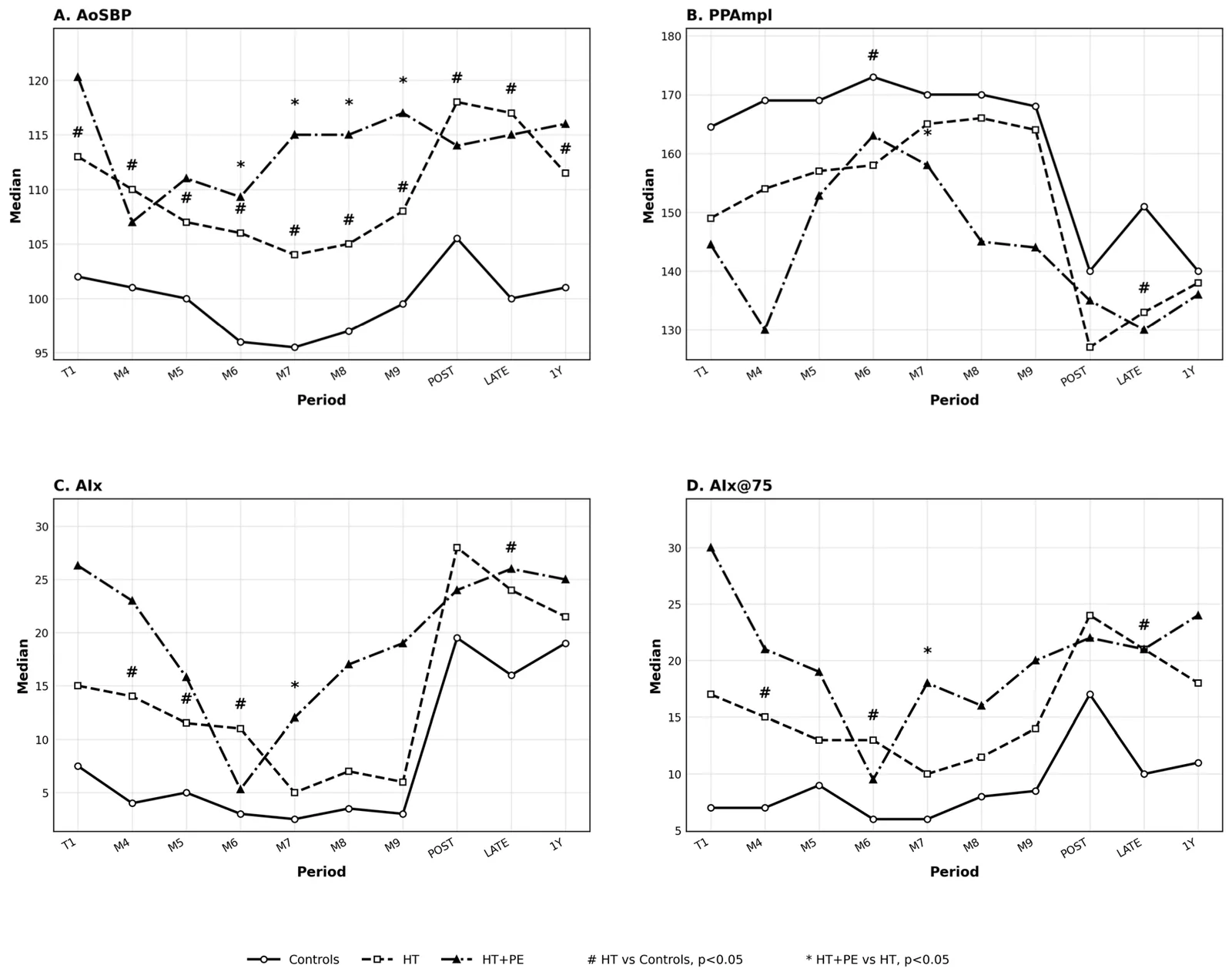
Profiles of central pulse wave analysis parameters in normotensive pregnancies (controls), uncomplicated hypertensive pregnancies (HT), and hypertensive pregnancies complicated by preeclampsia (HT + PE). (**A**) Aortic systolic blood pressure (AoSBP); (**B**) pulse pressure amplification (PPAmpl); (**C**) augmentation index (AIx); (**D**) augmentation index normalized to a heart rate of 75 beats/min (AIxHR75). Significant between-group differences (*p* < 0.05) are marked with [#] for control vs. HT and [*] for HT vs. HT + PE.

**Figure 2:**
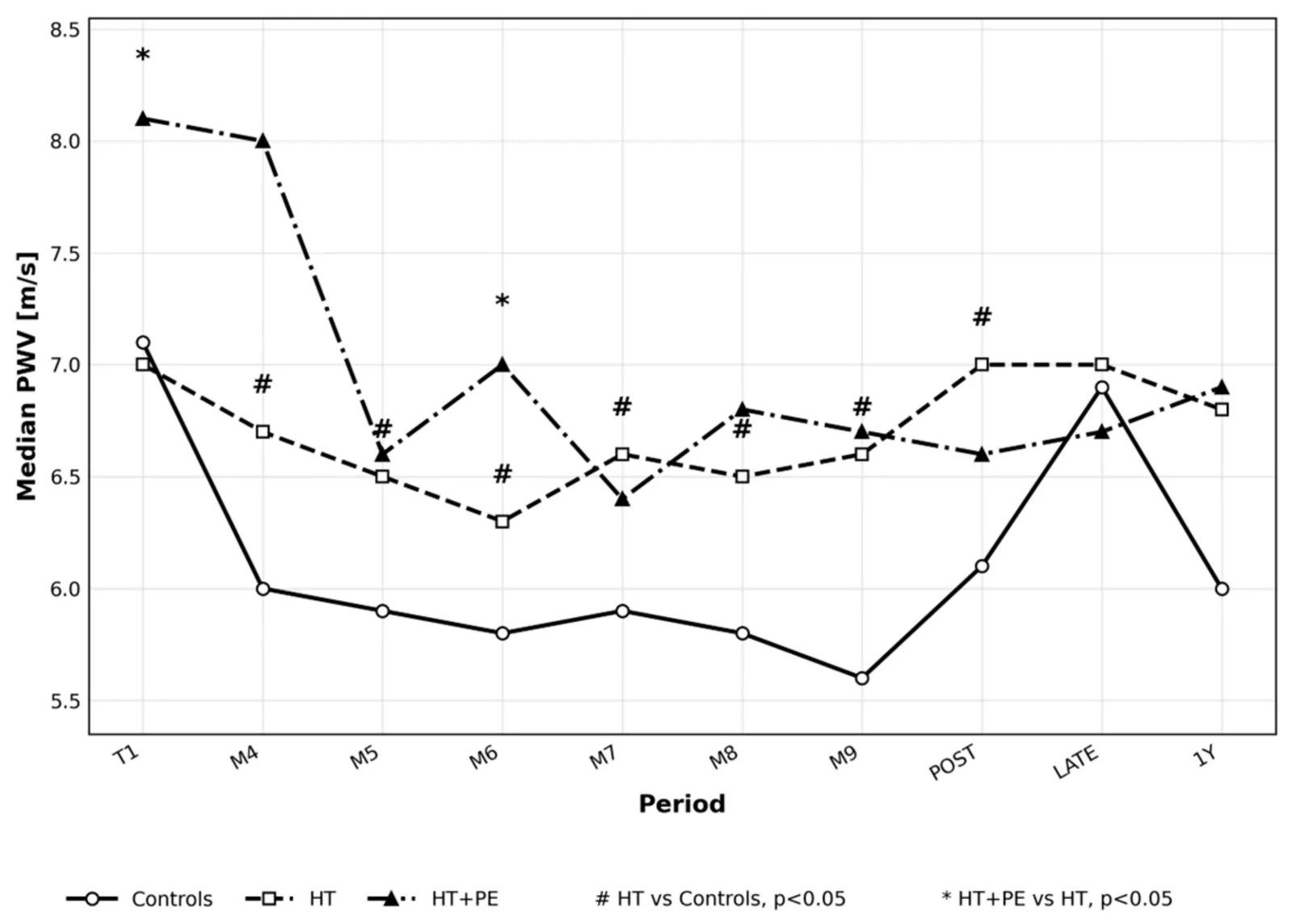
Profile of carotid–femoral pulse wave velocity (cfPWV) in normotensive pregnancies (controls), uncomplicated hypertensive pregnancies (HT), and hypertensive pregnancies complicated by preeclampsia (HT + PE). Significant between-group differences (*p* < 0.05) are marked with [#] for control vs. HT and [*] for HT vs. HT + PE.

The visualization of profiles of assessed pulse wave analysis parameters throughout pregnancy and postpartum and comparison in between the groups of patients are presented in Figure 1 (1A for AoSBP, 1B for PPAmpl, 1C for AIx and 1D for AIxHR75) and cfPWV in Figure 2.

Peripheral brachial blood pressure and central aortic blood pressure were significantly lower in normotensives (control group) than in both hypertensive groups—uncomplicated (HT) and preeclamptic (HT + PE) through whole pregnancy (*p*< 0.05), even though mean brachial BP was maintained below <140/90 mmHg in hypertensives with treatment managed in accordance with ESH/ESC guidelines for pregnancy [12,13], which was proven in ABPM assessment done simultaneously on each visit in examined population. The results of thorough peripheral blood pressure assessment, with office, home and 24 h ABPM measurements in the assessed population are presented elsewhere [18].

AoSBP showed the clearest separation between hypertensive women who remained uncomplicated and those who developed preeclampsia. Compared with the HT group, the HT + PE group had higher AoSBP from 23–26 weeks of gestation (M6) onward, with the largest numerical difference observed at 27–30 weeks (M7), and the difference persisted until delivery. These findings indicate group-level discrimination between hypertensive pregnancies with and without preeclampsia, but they should not be interpreted as validated individual prediction.

AIx and AIxHR75 tended to be lower, and PPAmpl tended to be higher, in controls than in hypertensive women during pregnancy. Significant control versus HT differences were observed mainly at 23–26 weeks for AIx/AIxHR75 and PPAmpl and additionally at 15–18 weeks for AIx/AIxHR75. Within hypertensive pregnancies, AIx/AIxHR75 and PPAmpl showed opposite directional changes after 23–26 weeks in the HT + PE group: augmentation indices increased, whereas PPAmpl decreased. Because no formal changepoint analysis was performed, this pattern is described as an observed change in trajectory rather than a turning point.

Comparisons between HT and HT + PE showed significant differences in AIx/AIxHR75 and PPAmpl mainly at 27–30 weeks (M7). Thereafter, AIx/AIxHR75 remained higher and PPAmpl lower in HT + PE, whereas values in uncomplicated hypertension were closer to those observed in controls. These findings suggest altered wave-reflection and pressure-amplification profiles in HT + PE, so at least a month before average time of PE recognition in the examined population, which was in 32.8 gestational week, although the interpretation of these indices must remain cautious.

cfPWV was lower in controls than in hypertensive women from 15 weeks of gestation until delivery and at 6–8 weeks postpartum. Within hypertensive pregnancies, cfPWV was numerically higher in the HT + PE group than in the HT group during most of pregnancy, with significant differences in the first trimester and at 23–26 weeks. Thus, cfPWV provided complementary information to AoSBP by reflecting aortic stiffness rather than central pressure load.

**Figure 3:**
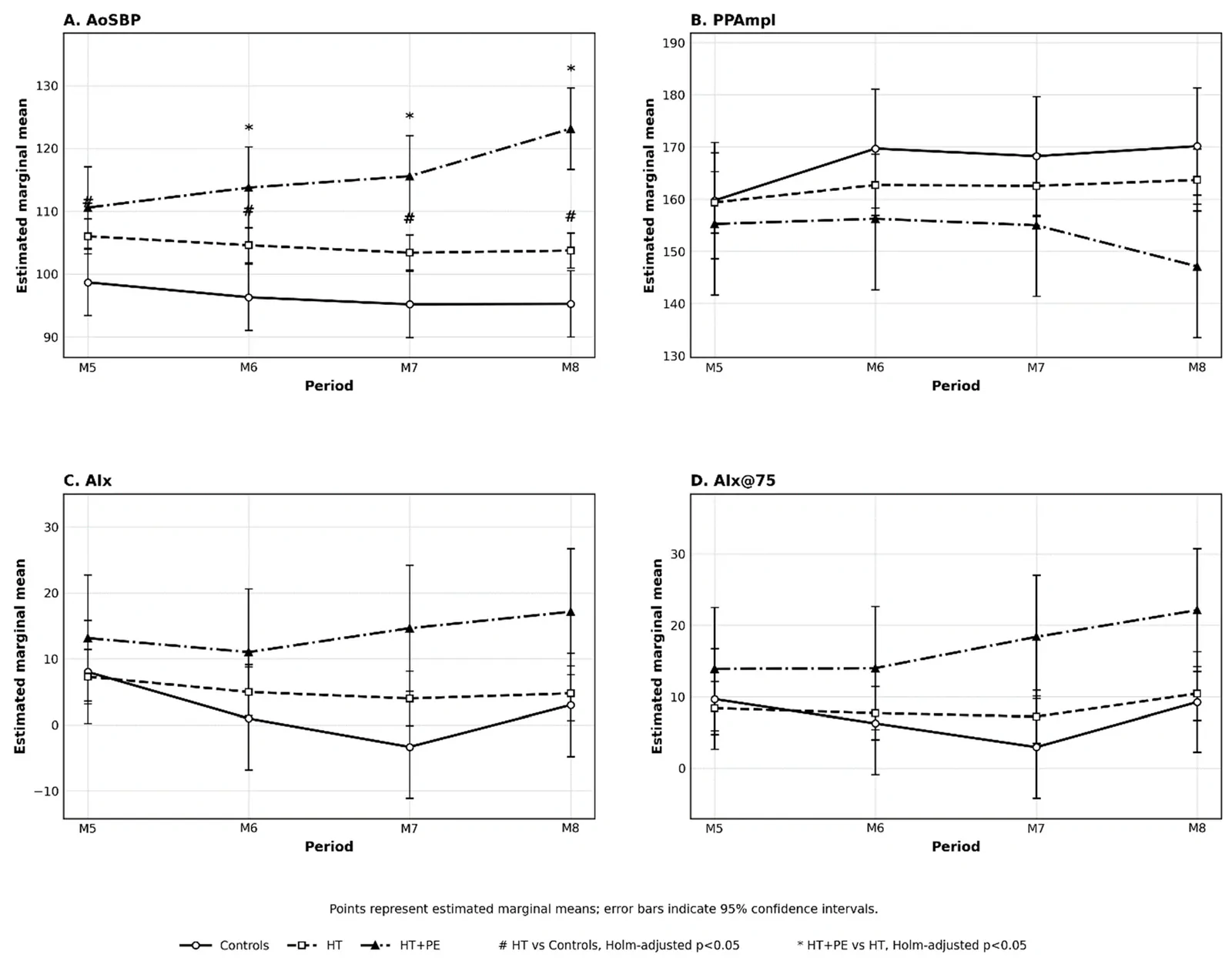
Profiles of pulse wave analysis parameters from M5 to M8 in normotensive pregnancies (controls), uncomplicated hypertensive pregnancies (HT), and hypertensive pregnancies complicated by preeclampsia (HT + PE). The analysis included patients with complete PWA and cfPWV observations at all time points from M5 to M8. (**A**) Aortic systolic blood pressure (AoSBP); (**B**) pulse pressure amplification (PPAmpl); (**C**) augmentation index (AIx); (**D**) augmentation index normalized to a heart rate of 75 beats/min (AIxHR75). Points represent estimated marginal means with 95% confidence intervals. Significant between-group differences after Holm adjustment (*p* < 0.05) are marked with [#] for controls vs. HT and [*] for HT vs. HT + PE.

**Figure 4:**
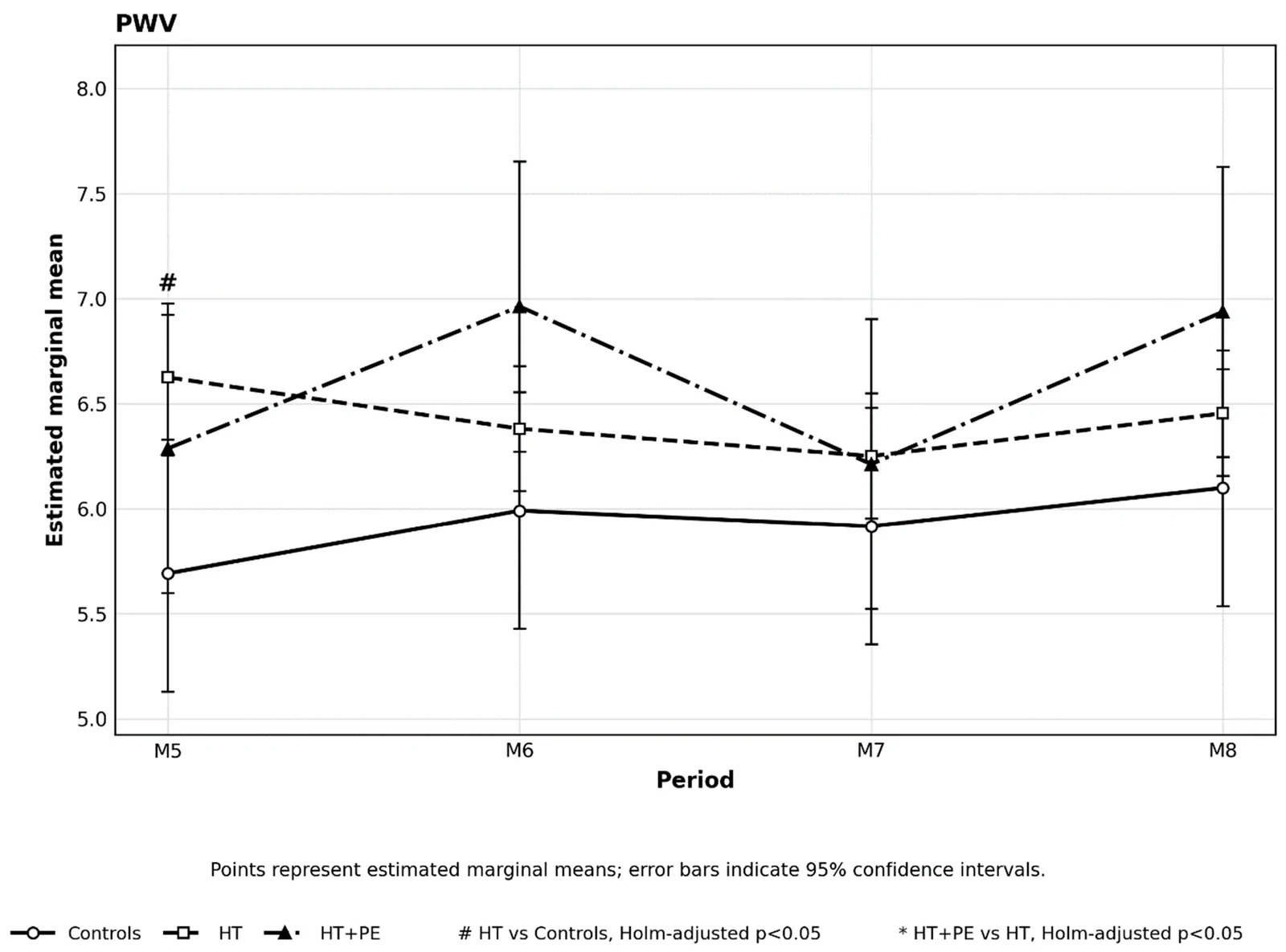
Profile of carotid–femoral pulse wave velocity (cfPWV) from M5 to M8 in normotensive pregnancies (controls), uncomplicated hypertensive pregnancies (HT), and hypertensive pregnancies complicated by preeclampsia (HT + PE). The analysis included patients with complete PWA and cfPWV observations at all time points from M5 to M8. Points represent estimated marginal means with 95% confidence intervals. Significant between-group differences after Holm adjustment (*p* < 0.05) are marked with [#] for controls vs. HT and [*] for HT vs. HT + PE.

A complete-case longitudinal analysis was then performed in women with available PWA and cfPWV observations at all four periods from M5 to M8. This subgroup included 63 women: 12 controls, 43 women with uncomplicated hypertension, and 8 women with HT + PE. Descriptive values for this subgroup are presented in Table 6, and mixed-effects model estimates are shown in Figure 3 and Figure 4.

For each parameter, a separate linear mixed-effects model was fitted with group, period, and the group × period interaction as fixed effects. EMMs with 95% confidence intervals were calculated for each group and period. Within-period comparisons were performed for control vs. HT and HT vs. HT + PE, with Holm adjustment for multiple comparisons. Adjusted *p* values < 0.05 were considered statistically significant; # denotes control vs. HT, and * denotes HT vs. HT + PE.

In the complete-case analysis, AoSBP was the PWA parameter that most clearly differentiated the groups. AoSBP was higher in HT than in controls across M5–M8, with differences of approximately 7–8 mmHg. In HT + PE, AoSBP was additionally higher than in HT from M6 onward, and the difference increased from approximately 9 mmHg at M6 to 19 mmHg at M8. The significant group × period interaction (*p* = 0.001) indicated different temporal trajectories between groups, mainly due to the progressive increase in AoSBP in HT + PE.

For PPAmpl, no significant effects of group, period, or their interaction were observed. AIx and AIxHR75 changed over time (*p* = 0.047 and *p* = 0.017, respectively), but no significant between-group differences or group-specific temporal patterns remained after Holm correction. These results indicate that AoSBP provided the most consistent group-level discrimination among the PWA parameters, whereas PPAmpl, AIx, and AIxHR75 showed weaker and less robust patterns. Interpretation of the latter parameters should remain cautious, particularly because the complete-case HT + PE subgroup was small (*n* = 8) and confidence intervals were wide.

**Figure 5:**
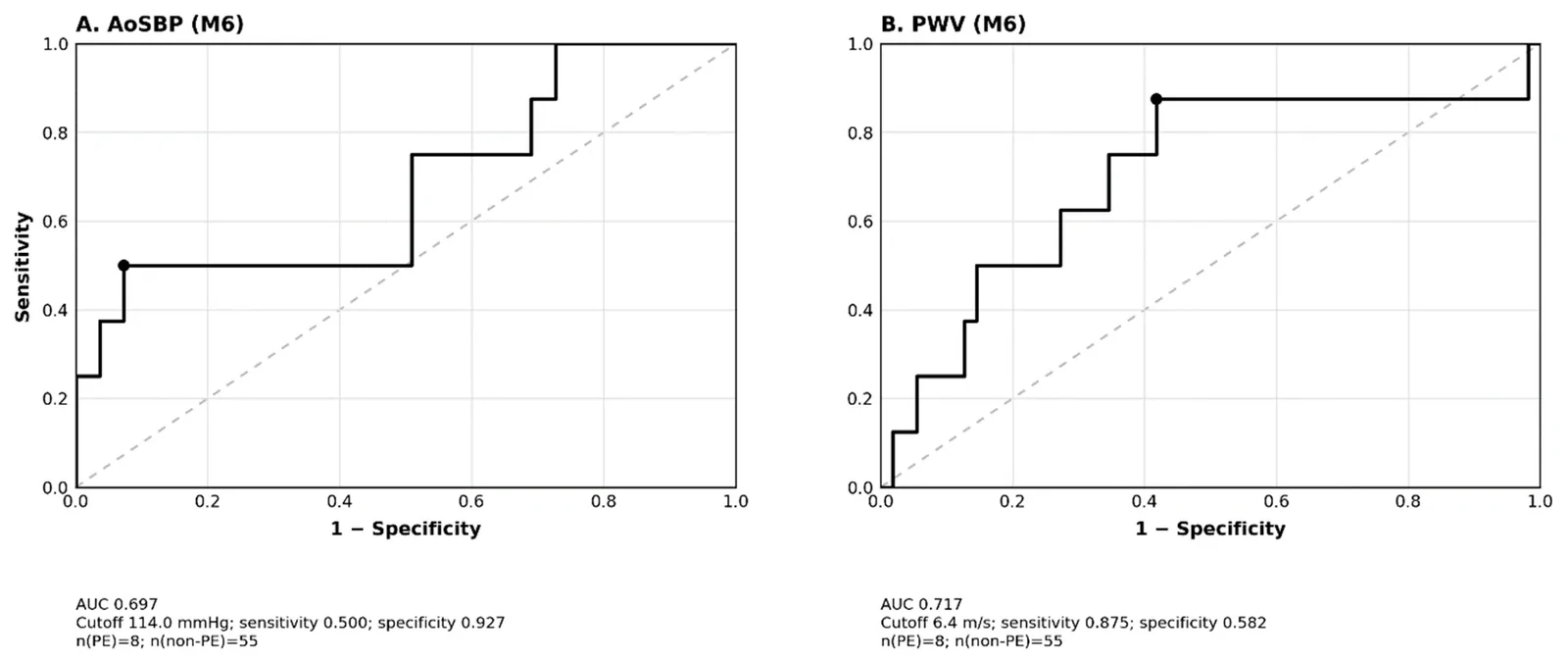
Receiver operating characteristic (ROC) curves evaluating the ability of vascular parameters measured at 23–26 weeks of gestation (M6) to discriminate pregnancies complicated by preeclampsia (PE) in the cohort with complete PWA and PWV observations from M5 through M8. (**A**) Aortic systolic blood pressure (AoSBP); (**B**) carotid–femoral pulse wave velocity (PWV). Black dots indicate the optimal cutoff values determined using the Youden index. The area under the curve (AUC), cutoff value, sensitivity, and specificity are provided below each panel. The analysis included 8 patients with PE and 55 patients without PE.

Exploratory ROC analyses were performed in the complete M5–M8 cohort to assess discrimination of pregnancies complicated by preeclampsia using vascular parameters measured at 23–26 weeks (M6). The analysis included 8 patients with preeclampsia and 55 patients without preeclampsia. AoSBP and cfPWV were selected because they represented complementary aspects of central pressure load and aortic stiffness. The ROC-derived cutoffs were obtained with the Youden index in the same cohort and therefore should be interpreted as hypothesis-generating rather than as clinically validated thresholds. The corresponding ROC curves are shown in Figure 5.

## 4. Discussion

The present prospective longitudinal study evaluated AoSBP, AIx, AIxHR75, PPAmpl, and cfPWV in normotensive pregnancy, uncomplicated hypertension in pregnancy, and hypertension complicated by preeclampsia from early pregnancy to one year postpartum. The main finding is that women with hypertension who developed preeclampsia showed a different central hemodynamic profile from women with uncomplicated hypertension. This profile was most evident for AoSBP, which increased progressively in HT + PE from mid-pregnancy, and was supported by selected differences in cfPWV. These results demonstrate an association between altered hemodynamic trajectories and preeclampsia-related pregnancy course; they yet do not establish clinically useful early prediction although suggest further studies in that field.

The profile observed in normotensive pregnancies is consistent with physiological vascular adaptation during uncomplicated gestation. Healthy pregnancy is characterized by vasodilation, lower systemic vascular resistance, and increased arterial compliance during the first half of pregnancy, followed by a partial return toward pre-pregnancy vascular tone near term. In this cohort, controls showed lower AoSBP, lower AIx/AIxHR75, and higher PPAmpl than hypertensive women for much of pregnancy, broadly in line with longitudinal and cross-sectional studies reporting a mid-pregnancy nadir in central pressure and wave-reflection indices [8,19,20,21].

In contrast, hypertensive pregnancies showed higher central aortic pressures despite treatment aimed at maintaining brachial blood pressure within the normal for general population values assessed in 24 h ABPM. This finding is clinically relevant because brachial blood pressure may not fully reflect central pressure load. Nevertheless, AoSBP is load-dependent and should be interpreted as an integrated hemodynamic signal rather than evidence of a blood-pressure-independent structural vascular abnormality. Previous adult studies have shown associations between central pressure and cardiovascular outcomes [6,7], and pregnancy studies have reported higher central pressure and arterial stiffness indices in hypertensive and preeclamptic pregnancies [10,11,22,23,24,25,26]. In the present cohort, AoSBP separated HT from HT + PE from 23–26 weeks onward at the group level.

AIx, AIxHR75, and PPAmpl provided complementary but less robust information. AIx is influenced by wave reflection, vascular tone, heart rate, body size, and arterial properties, and should not be treated as a direct measure of aortic stiffness. PPAmpl depends on the relationship between peripheral and central pulse pressure and can vary with gestational age and pressure load. The observed increase in AIx/AIxHR75 and decrease in PPAmpl in HT + PE after 23–26 weeks may indicate an altered integrated hemodynamic response, but these findings should not be interpreted as proof of a specific pathological vascular mechanism as pathophysiology of preeclampsia is more complex. Studies of pregnancy-associated hypertension have similarly reported impaired arterial adaptation and attenuation or reversal of the central-to-peripheral stiffness gradient [27,28,29].

The interpretation of AIx, AIxHR75, PPAmpl, and cfPWV in pregnancy therefore requires particular caution. Pregnancy involves dynamic changes in cardiac output, heart rate, arterial compliance, systemic vascular resistance, and vascular tone. As a result, divergent parameter trajectories may reflect a combined hemodynamic response rather than a specific cellular or structural mechanism. Because this study did not include endothelial function testing, angiogenic factors, placental biomarkers, inflammatory markers, or direct vascular-wall assessments, the observed differences cannot establish whether vascular alterations are causal, compensatory, or markers of broader maladaptation.

cfPWV is generally regarded as the most direct non-invasive measure of aortic stiffness among the parameters analyzed in this study. In the present cohort, cfPWV was lower in controls than in hypertensive women from 15 weeks until delivery and at 6–8 weeks postpartum. Within hypertensive pregnancies, cfPWV was higher in HT + PE during selected periods, including the first trimester and 23–26 weeks. These findings are consistent with previous studies showing increased PWV or pulse-wave-derived indices in women who subsequently developed preeclampsia [30,31], with longitudinal evidence of divergent arterial stiffness trajectories [32], and with prospective data indicating that arterial stiffness may contribute to preeclampsia risk assessment when considered with other clinical and biochemical markers [33]. Savvidou et al. reported higher carotid–femoral and carotid–radial PWV at 22–24 weeks in women who subsequently developed preeclampsia [31]. Phan et al. identified changepoints in cfPWV and crPWV at 14–17 weeks, followed by divergence of cfPWV trajectories between women who did and did not develop preeclampsia [32]. In a later prospective cohort, first-trimester arterial stiffness predicted preeclampsia with better discriminatory ability than peripheral blood pressure, uterine artery Doppler, or angiogenic biomarkers alone [33]. A recent systematic review and meta-analysis also reported elevated PWV in pregnancy-associated diseases, including preeclampsia and hypertension [34]. These observations are consistent with the present data, particularly the M6 cfPWV performance in ROC analysis.

The ROC analyses provide a cautious view of clinical applicability. The assessed parameters showed, at best, moderate discrimination, and the ROC thresholds were derived and evaluated in the same relatively small cohort using the Youden index. This design may overestimate diagnostic performance and does not allow the cutoffs to be used as clinical thresholds. Moreover, the analysis did not test incremental predictive value beyond clinical risk factors, brachial blood pressure, ABPM, uterine artery Doppler, or angiogenic biomarkers. Accordingly, the ROC results should be considered exploratory and hypothesis-generating.

The distinction between association, discrimination, and prediction is central to interpreting these results. The study demonstrates group-level differences in longitudinal central hemodynamic profiles and exploratory discrimination between HT and HT + PE in selected gestational windows. It does not demonstrate validated individual-level prediction of future preeclampsia. In addition, because preeclampsia was diagnosed between 20 and 39 weeks, some measurements in the mid-gestation windows may have been obtained close to or after clinical diagnosis in individual women. Therefore, the findings are described as altered trajectories associated with HT + PE rather than as proof that the measurements predict subsequent disease.

The prospective design and repeated assessments across pregnancy and postpartum are important strengths. They allowed evaluation of trajectories rather than isolated cross-sectional measurements and enabled comparison of normotensive pregnancies, uncomplicated hypertensive pregnancies, and hypertensive pregnancies complicated by preeclampsia. This comparison is clinically relevant because differentiating treated hypertension that remains uncomplicated from hypertension that progresses to preeclampsia is a common challenge in antenatal care. Previous protocols and reviews have emphasized the need for longitudinal assessment to clarify the role of central aortic pressure and arterial stiffness in hypertensive disorders of pregnancy [35,36].

The findings may also be considered within a broader framework of vascular-wall biology and vascular remodeling. Arterial mechanical behavior is influenced by vascular smooth-muscle cell function, extracellular-matrix turnover, metalloproteinase activity, inflammatory signaling, cytokine-mediated pathways, and mechanotransduction. Costa et al. reviewed these processes in the context of arterial aneurysmal disease, emphasizing their role in vascular-wall homeostasis and remodeling [37]. Although aneurysmal disease differs substantially from hypertensive disorders of pregnancy, this conceptual framework is useful for distinguishing the hemodynamic phenotype observed in this cohort from the underlying cellular and structural mechanisms that cannot be directly inferred from pulse-wave analysis alone.

Several limitations should be acknowledged. First, the study was observational, and causal inferences cannot be made. Second, although the total number of visits exceeded 1200, group sizes were unequal, and the number of women available at individual time points varied because of the real-life registry design, late study inclusion, earlier delivery, or clinical deterioration. Third, the complete-case mixed-effects and ROC analyses included only 8 women with HT + PE, resulting in wide confidence intervals and limited precision. Fourth, the hypertensive cohort included both chronic and gestational hypertension, which may differ in baseline vascular phenotype and in their relationship with preeclampsia. Fifth, antihypertensive treatment was individualized for clinical and ethical reasons and may have influenced central pressure, wave reflection, and arterial stiffness. Sixth, ROC analyses were exploratory, internally derived, and not externally validated. Finally, no direct biomarkers of endothelial function, placental dysfunction, angiogenic imbalance, inflammation, or vascular-wall remodeling were measured.

The study also has strengths. It used a prospective longitudinal design, repeated standardized measurements performed by a single trained investigator, assessment of central pressure, wave-reflection indices, PPAmpl, and cfPWV, and follow-up extending to one year postpartum. Inclusion of normotensive, uncomplicated hypertensive, and HT + PE groups allowed separation of the hemodynamic burden of hypertension from the additional phenotype associated with preeclampsia.

Future studies should determine whether serial central hemodynamic assessment improves risk classification beyond established clinical variables, 24-h blood pressure monitoring, uterine artery Doppler, and angiogenic biomarkers. Predefined multivariable models with external validation are needed before these measurements can be incorporated into routine antenatal risk stratification. Further work should also examine whether trajectories differ between chronic hypertension, gestational hypertension, early-onset preeclampsia, late-onset preeclampsia, and preeclampsia with fetal growth restriction.

## 5. Conclusions

In this prospective longitudinal cohort, central hemodynamic profiles differed between normotensive pregnancies, uncomplicated hypertensive pregnancies, and hypertensive pregnancies complicated by preeclampsia. AoSBP showed the clearest and most consistent group-level separation, whereas cfPWV provided complementary information on aortic stiffness. Women who developed preeclampsia showed higher AoSBP from 23–26 weeks of gestation onward, higher cfPWV during selected periods of pregnancy, and divergent trajectories of AIx/AIxHR75 and PPAmpl after mid-pregnancy. AIx, AIxHR75, and PPAmpl showed less robust between-group discrimination and should be interpreted as integrated hemodynamic indices rather than direct markers of a specific vascular mechanism. These findings suggest that impaired central vascular adaptation may precede the clinical recognition of preeclampsia in hypertensive pregnancies.

Overall, the findings identify a central hemodynamic phenotype associated with preeclampsia within hypertensive pregnancy. They do not establish clinically useful early prediction or causality. Exploratory ROC analyses showed only moderate discrimination for selected parameters, and the derived cutoffs require external validation. Serial central hemodynamic assessment may be valuable in future multivariable risk models, but external validation is required before clinical implementation and it should not currently be used as a stand-alone screening approach.

## Figures and Tables

**Table 1:** Basic characteristics of the studied population.

	All (*n* = 241)	Normotensive Control Group (*n* = 40)	Uncomplicated Hypertensive Patients (HT Group) (*n* = 152)	Hypertensive Patients with Preeclampsia (HT + PE Group) (*n* = 49)
Age, years	34 (31; 38)	33 (30; 35)	34 (30.5; 38)	36 (31; 38)
BMI before pregnancy, kg/m^2^	27.2 (22.2; 32)	27.2	27.6 (22.7; 31.9)	26.3 (20.8; 32)
Obesity (pre-pregnancy BMI ≥ 30 kg/m^2^); *n*, %	76, 31.5%	15, 37.5%	47, 30.9%	14, 28.6%
No. of kg gained in pregnancy	14 (9; 18)	13.5 (10; 15.5)	14 (9; 19)	12 (9; 18)
Hyperlipidemia; *n*, %	31, 12.9%	4, 10%	16, 10.5%	11, 22.4%
Pregestational glucose level abnormalities (IFG, IGT, DM); *n*, %	45, 18.7%	5, 12.5%	31, 20.4%	9, 18.4%
Gestational diabetes; *n*, %	74, 30.7%	9, 22.5%	50, 32.9%	15, 30.6%
Serum creatinine level, mg/dl	0.64 (0.58; 0.72)	0.62 (0.57; 0.7)	0.63 (0.58; 0.71)	0.69 (0.61; 0.8)
Nulliparity; *n*, %	90, 37.3%	12, 30%	53, 34.9%	25, 51%
Multiparity; *n*, %	151, 62.7%	28, 70%	99, 65.1%	24, 49%
Smoking; *n*, %	65, 27%	10, 25%	45, 29.6%	10, 20.4%
ASA in pregnancy; *n/N* %	199, 82.6%	37, 92.5%	132, 86.8%	30, 61.2%
Time of delivery; weeks of gestation	38 (37; 39)	38.5 (38; 40)	38 (38; 39)	37 (35; 38)
Premature birth; *n*, %	36, 14.9%	3, 7.5%	13, 8.5%	20, 40.8%
Cesarean section; *n*, %	132, 54.8%	17, 42.5%	75, 49.3%	40, 81.6%
Newborn’s body mass at birth; gr.	3090 (2530; 3480)	3355 (2955; 3740)	3220 (2833; 3500)	2320 (1700; 2640)
FGR; *n*, %	51, 21.2%	2, 5%	16, 10.5%	33, 67.3%
Apgar points at 1 min.	10 (9; 10)	10 (10; 10)	10 (10; 10)	10 (8; 10)
Apgar points at 10 min.	10 (10; 10)	10 (10; 10)	10 (10; 10)	10 (9; 10)
Newborn’s female gender; *n*, %	107, 44.4%	12, 30%	66, 43.4%	29, 59.2%
Miscarriage/IUD/newborn’s death; *n*, %	8, 3.3%	1, 2.5%	5, 3.3%	2, 4.1%

Abbreviations: ASA, acetylsalicylic acid; BMI, body mass index; DM, diabetes mellitus type 1 or type 2; FGR, fetal growth retardation; IFG, impaired fasting glucose, IGT, impaired glucose tolerance; IUD, intrauterine death.

**Table 2:** Number and percentage of patients participating in each visit of the study.

	Normotensive Control Group (*n* = 40)	Uncomplicated Hypertensive Patients (HT Group) (*n* = 152)	Hypertensive Patients with Preeclampsia (HT + PE Group) (*n* = 49)
<15 w.g. (T1)	11, 27.5%	53, 34.9%	9, 18.4%
15–18 w.g. (M4)	22, 55%	86, 56.6%	11, 22.5%
19–22 w.g. (M5)	26, 65%	87, 57.2%	17, 34.7%
23–26 w.g. (M6)	23, 57.5%	97, 63.8%	19, 38.8%
27–30 w.g. (M7)	26, 65%	101, 66.5%	22, 44.9%
31–34 w.g. (M8)	27, 67.5%	100, 65.8%	22, 44.9%
35–41 w.g. (M9)	16, 40%	80, 52.6	13, 26.5%
6–8 weeks postpartum (POST)	9, 22.5%	89, 58.6%	33, 67.4%
6 months postpartum (LATE)	5, 12.5%	64, 42.1%	31, 63.3%
>12 months postpartum (1Y)	5, 12.5%	44, 29%	21, 42.9%

**Table 3:** Peripheral and central hemodynamic parameters in normotensive pregnancies. Data are presented as medians (Q1; Q3).

	pSBP [mmHg]	pDBP [mmHg]	HR [Beats/min.]	AoSBP [mmHg]	AoDBP [mmHg]	AIx [%]	AIxHR75 [%]	PPAmpl [%]	cfPWV [m/s]
<15 w.g. (T1)	122.5 (116.2; 133.5)	71 (66.9; 76.8)	78 (67.8; 80)	102 (99.2; 110.8)	72 (68.8; 78.5)	7.5 (1.5; 18.1)	7 (3.2; 19.8)	164.5 (140.9; 175.2)	7.1 (6.3; 7.3)
15–18 w.g. (M4)	115 (110; 123)	67 (63; 72)	75 (70; 81)	101 (94; 102)	68 (64; 73)	4 (−2; 12)	7 (−1; 11)	169 (147; 177)	6 (5.7; 6.7)
19–22 w.g. (M5)	117 (115; 122)	67 (64; 71)	80 (73; 88)	100 (96; 102)	69 (65; 74)	5 (2; 11)	9 (1; 16)	169 (153; 173)	5.9 (5.5; 6.6)
23–26 w.g. (M6)	114.5 (107.8; 118)	68 (64.; 70)	81 (76.8; 90.2)	96 (94.5; 99.6)	70 (65.8; 72.1)	3 (−4.2; 8.2)	6 (−0.5; 13)	173 (161; 178.5)	5.8 (5.2; 6.7)
27–30 w.g. (M7)	111 (107.2; 115)	67.5 (63.5; 70.8)	85 (74.2; 95.8)	95.5 (93; 99)	69 (65.2; 72.8)	2.5 (−6; 8.8)	6 (−1; 12)	170 (158; 179)	5.9 (5.3; 6.8)
31–34 w.g. (M8)	111 (106.2; 117.2)	70 (64.5; 75)	84.5 (77.2; 96.8)	97 (92; 101.8)	71.5 (66.5; 76)	3.5 (−4; 12.8)	8 (4; 16)	170 (157; 178)	5.8 (5.3; 6.7)
35–41 w.g. (M9)	111 (103.5; 118.5)	69.5 (65.5; 75)	88.5 (78.5; 96)	99.5 (90; 101.5)	71 (68; 76)	3 (−2.5; 9.5)	8.5 (2.2; 15.5)	168 (152.5; 178.5)	5.6 (5.3; 6.3)
6–8 weeks postpartum (POST)	116 (114; 119)	71 (67.8; 72)	65.5 (56.5; 67)	105.5 (102.8; 108.2)	72 (69; 73)	19.5 (18.8; 30)	17 (14; 20.8)	140 (127; 143.2)	6.1 (5.8; 6.4)
6 months postpartum (LATE)	116 (115; 117)	69 (63; 72)	66 (60; 74)	100 (100; 105)	70 (64; 73)	16 (11; 17)	10 (6; 13)	151 (141; 154)	6.9 (6.3; 7)
>12 months postpartum (1Y)	114 (113; 115)	68 (66; 70)	60 (56; 62)	101 (96; 102)	69 (67; 72)	19 (8; 22)	11 (8; 15)	140 (134; 160)	6 (6; 7)

Abbreviations: AIx, augmentation index; AIxHR75, augmentation index normalized to a heart rate of 75 beats per minute; AoDBP aortic diastolic blood pressure; AoSBP, aortic systolic blood pressure; HR, heart rate; pDBP, peripheral brachial diastolic blood pressure; PPAmpl, pulse pressure amplification; pSBP, peripheral brachial systolic blood pressure; cfPWV, carotid-femoral pulse wave velocity; Q1, first quartile, Q3, third quartile; w.g., weeks of gestation.

**Table 4:** Peripheral and central hemodynamic parameters in uncomplicated hypertensive pregnancies (HT group). Data are presented as medians (Q1; Q3).

	pSBP [mmHg]	pDBP [mmHg]	HR [Beats/min.]	AoSBP [mmHg]	AoDBP [mmHg]	AIx [%]	AIxHR75 [%]	PPAmpl [%]	cfPWV [m/s]
<15 w.g. (T1)	132 (126; 136.5)	76.7 (71; 84.5)	78 (70; 83)	113 (110; 122)	79 (72; 86)	15 (11; 22)	17 (10; 25)	149 (134; 156)	7 (6.4; 7.4)
15–18 w.g. (M4)	128 (119; 134.5)	74 (67.5; 83.5)	77 (70; 83.5)	110 (103; 117)	76 (69; 85)	14 (5; 21)	15 (5; 21.5)	154 (139; 167.5)	6.7 (6.1; 7.3)
19–22 w.g. (M5)	123 (117.8; 128)	73 (67; 80.5)	79 (72; 86.6)	107 (101.8; 112)	74.5 (68; 83)	11.5 (4; 21)	13 (3; 21.2)	157 (143; 172.2)	6.5 (6; 7.7)
23–26 w.g. (M6)	123 (116; 128)	73 (67; 79)	81 (75; 88.5)	106 (99.5; 111.5)	74 (69; 80.5)	11 (2; 17)	13 (2; 20)	158 (148.5; 172.8)	6.3 (6; 7.1)
27–30 w.g. (M7)	120 (114.5; 127)	74 (68.5; 79)	84 (78; 93.5)	104 (98; 109)	76 (71; 80)	5 (−2.8; 14.5)	10 (2; 19.2)	165 (150; 179)	6.6 (5.8; 7.3)
31–34 w.g. (M8)	120 (115; 127)	75 (71; 82)	86 (79; 95)	105 (99; 109)	77 (72; 84)	7 (−4; 14)	11.5 (5; 19)	166 (149; 177)	6.5 (6; 7.2)
35–41 w.g. (M9)	121.5 (117; 129)	78 (73; 83)	88 (80; 95)	108 (102; 113)	79 (74.5; 86)	6 (−1; 18)	14 (6.5; 22)	164 (144; 178.2)	6.6 (5.8; 7)
6–8 weeks postpartum (POST)	126 (121; 135)	75 (70; 83)	68 (60; 74.8)	118 (111; 125)	77 (71; 85)	28 (22; 33.8)	24 (18; 29)	127 (117.2; 138)	7 (6.4; 7.8)
6 months postpartum (LATE)	127 (121; 136)	77 (70; 83)	69 (62; 76.5)	117 (108.5; 124)	78 (71; 84.5)	24 (17; 30.5)	21 (12.5; 27)	133 (121.5; 144.5)	7 (6.2; 7.7)
>12 months postpartum (1Y)	124 (119.8; 135.2)	75.5 (68.8; 84.2)	67 (61.8; 74)	111.5 (106.5; 123)	76.5 (70; 85.5)	21.5 (16.8; 26)	18 (13; 24)	138 (127; 144.5)	6.8 (6.2; 7.7)

Abbreviations: AIx, augmentation index; AIxHR75, augmentation index normalized to a heart rate of 75 beats per minute; AoDBP aortic diastolic blood pressure; AoSBP, aortic systolic blood pressure; HR, heart rate; pDBP, peripheral brachial diastolic blood pressure; PPAmpl, pulse pressure amplification; pSBP, peripheral brachial systolic blood pressure; cfPWV, carotid-femoral pulse wave velocity; Q1, first quartile, Q3, third quartile; w.g., weeks of gestation.

**Table 5:** Peripheral and central hemodynamic parameters in hypertensive pregnancies complicated by preeclampsia (HT + PE group). Data are presented as medians (Q1; Q3).

	pSBP [mmHg]	pDBP [mmHg]	HR [Beats/min.]	AoSBP [mmHg]	AoDBP [mmHg]	AIx [%]	AIxHR75 [%]	PPAmpl [%]	cfPWV [m/s]
<15 w.g. (T1)	134.2 (121.8; 142.9)	87 (77.5; 92.8)	79 (65.2; 90.5)	120.2 (111.2; 133.4)	89.2 (78.8; 95.1)	26.2 (15.8; 30.4)	30 (14.2; 31.8)	144.5 (133.8; 176.2)	8.1 (7.8; 8.3)
15–18 w.g. (M4)	125 (117; 135)	75 (67; 81.5)	69 (63; 96)	107 (104; 123)	76 (70; 83.5)	23 (11; 34)	21 (11; 31)	130 (120; 164)	8 (6.6; 8.6)
19–22 w.g. (M5)	124 (117; 140.8)	75.5 (68.1; 88.6)	76.2 (69; 90)	111 (102.5; 133.5)	77.5 (69.2; 90.4)	15.8 (4.8; 26.8)	19 (6; 24)	152.8 (127.5; 177.2)	6.6 (6; 7.2)
23–26 w.g. (M6)	127 (125.2; 133.5)	77.5 (72.2; 82.8)	83.5 (72.8; 90.8)	109.2 (105.5; 118)	78.5 (75; 84)	5.2 (−0.5; 21.9)	9.5 (4; 26)	163 (137; 173)	7 (6.6; 7.9)
27–30 w.g. (M7)	130 (125; 135)	79 (72; 86)	82 (77; 91)	115 (105; 123)	81 (74; 87)	12 (6; 24)	18 (9; 27)	158 (132; 169)	6.4 (6; 7.4)
31–34 w.g. (M8)	127 (123; 134)	80 (74; 86)	84 (70; 94)	115 (106; 118)	81 (76; 88)	17 (−1; 26)	16 (5; 27)	145 (132; 180)	6.8 (6; 8.2)
35–41 w.g. (M9)	129 (127; 132)	84 (82; 88)	80 (75; 86)	117 (110; 124)	85 (83; 89)	19 (9; 25)	20 (15; 25)	144 (131; 170)	6.7 (6.1; 7.9)
6–8 weeks postpartum (POST)	125 (120; 132)	80 (71; 86)	72 (66; 77)	114 (107; 123)	81 (71; 88)	24 (20; 29)	22 (19; 27)	135 (125; 142)	6.6 (6.3; 7.4)
6 months postpartum (LATE)	128 (117; 132)	76 (71; 83)	71 (63; 79)	115 (105; 124)	77 (74; 84)	26 (17; 31)	21 (17; 29)	130 (125; 145)	6.7 (6.2; 7.2)
>12 months postpartum (1Y)	129 (116; 134)	79 (71; 83)	71 (62; 76)	116 (109; 125)	80 (72; 85)	25 (17; 33)	24 (15; 27)	136 (119; 147)	6.9 (6.5; 7.5)

Abbreviations: AIx, augmentation index; AIxHR75, augmentation index normalized to a heart rate of 75 beats per minute; AoDBP aortic diastolic blood pressure; AoSBP, aortic systolic blood pressure; HR, heart rate; pDBP, peripheral brachial diastolic blood pressure; PPAmpl, pulse pressure amplification; pSBP, peripheral brachial systolic blood pressure; cfPWV, carotid-femoral pulse wave velocity; Q1, first quartile, Q3, third quartile; w.g., weeks of gestation.

**Table 6:** Peripheral and central hemodynamic parameters assessed by pulse wave analysis and carotid–femoral pulse wave velocity in patients with complete PWA and cfPWV observations from M5 through M8, stratified by study group. Data are presented as medians (Q1; Q3).

	pSBP [mmHg]	pDBP [mmHg]	HR [Beats/min.]	AoSBP [mmHg]	AoDBP [mmHg]	AIx [%]	AIxHR75 [%]	PPAmpl [%]	cfPWV [m/s]
Controls (*n* = 12)									
19–22 w.g. (M5)	115.5 (112.5; 118.2)	66.0 (63.0; 68.5)	80.5 (71.0; 84.2)	98.5 (95.5; 102.0)	67.0 (64.8; 72.0)	5.5 (1.0; 15.0)	8.0 (1.5; 18.0)	162.5 (149.8; 172.8)	5.6 (5.3; 5.9)
23–26 w.g. (M6)	116.5 (106.8; 118.8)	68.0 (64.0; 70.0)	81.0 (76.2; 94.0)	97.0 (94.5; 100.0)	70.0 (65.8; 72.1)	−0.5 (−5.5; 6.4)	5.0 (−2.0; 12.5)	176.0 (168.0; 180.5)	5.8 (5.2; 6.6)
27–30 w.g. (M7)	111.5 (109.8; 115.0)	67.0 (64.2; 69.2)	88.0 (75.8; 97.2)	94.0 (93.0; 99.2)	68.0 (65.2; 70.5)	−1.5 (−19.5; 7.5)	3.0 (−9.5; 10.0)	171.0 (166.0; 183.5)	5.5 (5.3; 6.3)
31–34 w.g. (M8)	111.5 (105.0; 113.5)	67.5 (62.0; 75.0)	90.0 (76.8; 101.2)	95.5 (90.8; 98.8)	71.0 (63.8; 76.0)	3.5 (0.0; 13.2)	9.5 (3.0; 17.2)	169.5 (162.5; 183.0)	5.6 (5.1; 6.4)
HT (*n* = 43)									
19–22 w.g. (M5)	122.0 (117.5; 127.0)	72.0 (67.0; 78.0)	76.0 (71.5; 83.0)	106.0 (101.0; 109.5)	73.0 (68.0; 80.0)	9.0 (−0.5; 15.5)	7.0 (0.0; 17.0)	159.0 (147.5; 174.5)	6.2 (5.7; 7.3)
23–26 w.g. (M6)	123.0 (116.5; 127.5)	72.0 (67.0; 77.0)	79.0 (72.5; 85.5)	104.0 (98.5; 109.5)	73.0 (69.0; 78.0)	4.0 (−4.5; 14.0)	8.0 (−1.0; 17.0)	166.0 (152.0; 176.0)	6.3 (5.8; 6.8)
27–30 w.g. (M7)	120.0 (113.0; 127.0)	72.0 (67.5; 78.0)	80.0 (74.5; 90.0)	103.0 (97.5; 108.5)	73.0 (69.5; 79.0)	4.0 (−4.5; 13.0)	7.0 (0.2; 14.5)	167.5 (150.0; 176.0)	6.1 (5.7; 6.8)
31–34 w.g. (M8)	119.0 (115.0; 123.8)	74.0 (69.2; 78.5)	84.0 (78.5; 93.0)	104.0 (99.0; 107.5)	75.0 (71.0; 79.5)	5.0 (−6.0; 13.5)	11.0 (5.0; 19.0)	164.0 (154.0; 176.0)	6.2 (5.8; 6.9)
HT + PE (*n* = 8)									
19–22 w.g. (M5)	123.5 (114.8; 128.2)	68.8 (66.5; 80.1)	76.0 (71.0; 79.9)	103.0 (97.5; 120.8)	72.5 (67.8; 81.9)	13.2 (−4.2; 28.4)	12.5 (1.8; 25.0)	152.8 (127.8; 183.0)	6.2 (5.9; 6.6)
23–26 w.g. (M6)	126.5 (122.5; 141.2)	73.5 (70.5; 84.0)	83.5 (70.0; 88.0)	108.5 (102.0; 122.5)	76.0 (72.5; 86.0)	4.5 (−5.5; 29.5)	10.0 (2.0; 29.0)	155.5 (132.8; 182.5)	7.0 (6.5; 7.6)
27–30 w.g. (M7)	128.0 (124.8; 141.0)	79.0 (71.0; 85.2)	83.5 (75.6; 91.4)	117.0 (104.8; 128.2)	81.5 (73.2; 87.2)	16.8 (9.0; 23.2)	20.0 (8.0; 27.2)	153.8 (132.8; 166.5)	6.1 (5.9; 6.4)
31–34 w.g. (M8)	128.0 (125.2; 143.0)	86.5 (81.2; 91.0)	88.5 (79.0; 94.5)	119.0 (112.5; 133.5)	88.5 (82.5; 93.5)	24.5 (12.5; 27.0)	26.5 (20.2; 30.2)	139.0 (132.0; 159.0)	7.0 (6.0; 7.5)

Abbreviations: AIx, augmentation index; AIxHR75, augmentation index normalized to a heart rate of 75 beats per minute; AoDBP aortic diastolic blood pressure; AoSBP, aortic systolic blood pressure; HR, heart rate; pDBP, peripheral brachial diastolic blood pressure; PPAmpl, pulse pressure amplification; pSBP, peripheral brachial systolic blood pressure; cfPWV, carotid-femoral pulse wave velocity; Q1, first quartile, Q3, third quartile; w.g., weeks of gestation.

## Data Availability

The raw data supporting the conclusions of this article will be made available by the authors on request.

## Supplementary Materials

The following supporting information can be downloaded at: https://www.mdpi.com/article/10.3390/jcm15218247/s1, Supplementary Table S1. STROBE Statement checklist for cohort studies.

## Abbreviations

The following abbreviations are used in this manuscript:

ABPM	automated 24-h blood pressure monitoring
AIx	augmentation index
AIxHR75	augmentation index normalized to a heart rate of 75 beats per minute
AoDBP	aortic diastolic blood pressure
AoSBP	aortic systolic blood pressure
ASA	acetylsalicylic acid
BMI	body mass index
DM	diabetes mellitus
ESH/ESC	European Society of Hypertension/European Society of Cardiology
FGR	fetal growth retardation
HR	heart rate
HT group	group of patients with uncomplicated hypertension in pregnancy
HT + PE group	group of patients with preeclampsia in pregnancy
IFG	impaired fasting glucose
IGT	impaired glucose tolerance
IUD	intrauterine death
PE	preeclampsia
pDBP	peripheral (brachial) blood pressure
PPAmpl	pulse pressure amplification
pSBP	peripheral (brachial) blood pressure
PWV	pulse wave velocity
w.g.	weeks of gestation
