# Supplementary material for: Prospective Longitudinal Assessment of Central Hemodynamic Profile in Normotensive, Hypertensive and Preeclamptic Women in Pregnancy and Postpartum
Source: J Clin Med. 2026 Oct 23;15(21):8247. doi: 10.3390/jcm15218247 (PMC13763830; doi:10.3390/jcm15218247)
Supplement: Supplementary file 1 [file jcm-15-08247-s001.zip › jcm-4550101-supplementary.pdf]

Supplementary Table S1. STROBE Statement checklist for cohort studies

Prospective longitudinal assessment of central hemodynamic profile in normotensive, hypertensive and preeclamptic women in pregnancy and postpartum

Reporting guideline: STROBE Statement for observational cohort studies. This checklist was completed for a prospective longitudinal observational cohort study with repeated hemodynamic measurements during pregnancy and postpartum.

STROBE item	Recommendation for cohort studies	Location in manuscript	Status / comment for this study
**Title and abstract**			
**1a**	Indicate the study design in the title or abstract using a standard term.	Title; Abstract	Reported. The manuscript identifies the study as prospective, longitudinal, and observational.
**1b**	Provide a balanced abstract summarizing the study objective, methods, main findings, and interpretation.	Abstract	Reported. The revised abstract summarizes the cohort, methods, key hemodynamic findings, and cautions that the findings indicate group-level association/discrimination rather than validated early prediction.
**Introduction**			
**2**	Explain the scientific background and rationale.	Introduction	Reported. The manuscript describes hypertensive disorders of pregnancy, central hemodynamic assessment, and the need for longitudinal data.
**3**	State specific objectives and any prespecified hypotheses.	End of Introduction	Reported. The objective is to evaluate longitudinal profiles of AoSBP, AIx, AIxHR75, PPAmpl, and cfPWV in normotensive pregnancy, uncomplicated hypertension, and hypertension complicated by preeclampsia.
**Methods**			
**4**	Present the key elements of the study design early in the paper.	Materials and Methods, Section 2.1	Reported. The study is described as a prospective longitudinal observational study.
**5**	Describe the setting, location, study dates, recruitment period, follow-up, and data collection period.	Materials and Methods, Section 2.1	Reported. The manuscript states that pregnant women were followed at a tertiary outpatient clinic between May 2017 and October 2023, with pregnancy and postpartum follow-up.
**6a**	Give eligibility criteria, sources and methods of participant selection, and methods of follow-up.	Materials and Methods, Section 2.1	Reported. The manuscript describes separate eligibility criteria for chronic hypertension, pregnancy-induced/gestational hypertension, and normotensive controls; it also clarifies recruitment of hypertensive women and normotensive controls and reports follow-up visits.
**6b**	For matched studies, give matching criteria and numbers of exposed and unexposed participants.	Not applicable	Not applicable. This was not a matched cohort study.
**7**	Define outcomes, exposures, predictors, potential confounders, and effect modifiers.	Materials and Methods, Sections 2.1, 2.2, 2.5 and 2.6	Reported. The manuscript defines hypertension groups, preeclampsia, central hemodynamic parameters, and collected clinical variables. No confounder-adjusted multivariable prediction model was fitted.
**8**	For each variable of interest, give sources of data and details of assessment methods.	Materials and Methods, Sections 2.2–2.5	Reported. Office BP, ABPM-based hypertension classification, applanation tonometry, PWA, cfPWV, and clinical follow-up variables are described.
**9**	Describe efforts to address potential sources of bias.	Materials and Methods, Sections 2.1–2.4; Discussion, Limitations	Reported. Standardized measurement conditions, a validated device, and a single trained investigator were used. Remaining limitations include the single-center real-life registry design, unequal group sizes, treatment heterogeneity, and incomplete longitudinal data.
**10**	Explain how the study size was determined.	Materials and Methods, Section 2.1	Reported. No formal sample-size calculation was performed; the sample size was based on feasible recruitment in a real-life longitudinal registry.
**11**	Explain how quantitative variables were handled in the analyses.	Materials and Methods, Section 2.6; Results, Tables 1–6	Reported. Continuous variables are summarized as medians with quartiles; selected longitudinal analyses use estimated marginal means with 95% confidence intervals from mixed-effects models.
**12a**	Describe all statistical methods, including methods used to address confounding.	Materials and Methods, Section 2.6	Reported. The manuscript describes Mann–Whitney U tests, mixed-effects models, Holm adjustment, and exploratory ROC analyses. It states that incremental predictive value beyond conventional clinical variables and BP measurements was not assessed.
**12b**	Describe methods used to examine subgroups and interactions.	Materials and Methods, Section 2.6; Results, Figures 3–4	Reported. Group, period, and group × period interactions were examined in mixed-effects models in the complete M5–M8 subgroup.

**12c**	Explain how missing data were handled.	Materials and Methods, Section 2.6; Results, Tables 2 and 6	Reported. Visit-specific participation is shown, and complete-case analyses were restricted to women with available PWA and cfPWV observations at M5–M8. No imputation was reported.
**12d**	For cohort studies, explain how loss to follow-up was addressed.	Results, Table 2; Discussion, Limitations	Reported. Visit-level participation is presented. The manuscript acknowledges variable numbers at time points due to the real-life registry design, late inclusion, earlier delivery, or clinical deterioration.
**12e**	Describe sensitivity analyses, where applicable.	Not applicable / not performed	No sensitivity analyses were reported.
**Results**			
**13a**	Report numbers of individuals at each stage of the study.	Results; Tables 1, 2 and 6	Reported. The manuscript reports 241 participants, including 40 controls and 201 hypertensive women; 49 developed preeclampsia. Participation by study visit and the complete M5–M8 subgroup are reported.
**13b**	Give reasons for non-participation at each stage, where available.	Results, Table 2; Discussion, Limitations	Reported in part. Numbers at each visit are presented; reasons for missing visits are not fully itemized, but the limitations discuss the real-life registry design, late inclusion, earlier delivery, and clinical deterioration.
**13c**	Consider using a flow diagram.	Not included	A flow diagram is not included. Tables 1, 2, and 6 provide group numbers, visit participation, and complete-case subgroup size.
**14a**	Give characteristics of study participants and information on exposures and potential confounders.	Results, Table 1; Materials and Methods, Sections 2.1–2.2	Reported. Baseline and pregnancy characteristics include age, BMI, obesity, parity, smoking, diabetes, ASA use, delivery outcomes, neonatal outcomes, gestational age at consent, and antihypertensive treatment status at enrollment for the subgroup with medication data.
**14b**	Indicate the number of participants with missing data for each variable of interest.	Results, Tables 2 and 6	Reported. Visit-level availability and the complete-case analysis subgroup are reported.
**14c**	Summarize follow-up time.	Materials and Methods, Section 2.1; Results, Table 2	Reported. Follow-up included repeated pregnancy visits and postpartum visits at 6–8 weeks, 6 months, and 12 months.
**15**	Report the number of outcome events or summary measures over time.	Results	Reported. Preeclampsia developed in 49 hypertensive women. The manuscript reports gestational age at PE diagnosis and pregnancy/neonatal events.
**16a**	Give unadjusted and, where applicable, adjusted estimates with precision.	Results, Tables 1–6; Figures 1–5	Reported. The manuscript reports medians with quartiles and model-derived EMMs with 95% confidence intervals.
**16b**	Report category boundaries when continuous variables are categorized.	Materials and Methods, Section 2.6; Figure 5	Reported. ROC cutoffs were derived using the Youden index and are presented as exploratory internally derived thresholds.
**16c**	If relevant, translate relative risk into absolute risk.	Not applicable	Not applicable. The manuscript does not report relative risks or hazard ratios.
**17**	Report other analyses, including subgroup, interaction, and sensitivity analyses.	Materials and Methods, Section 2.6; Results, Table 6; Figures 3–5	Reported in part. Mixed-effects interaction analyses and exploratory ROC analyses are reported in the complete M5–M8 subgroup. No sensitivity analyses were reported.
**Discussion**			
**18**	Summarize key results with reference to study objectives.	Discussion	Reported. The Discussion summarizes longitudinal differences in AoSBP, AIx/AIxHR75, PPAmpl, and cfPWV across study groups.
**19**	Discuss limitations, including sources of bias, imprecision, and direction/magnitude of potential bias.	Discussion, Limitations	Reported. Limitations include observational design, unequal group sizes, small complete-case HT+PE subgroup, incomplete data, treatment heterogeneity, exploratory ROC analyses, lack of multivariable prediction modeling, and absence of external validation.
**20**	Provide a cautious overall interpretation considering objectives, limitations, multiplicity, and prior evidence.	Discussion; Conclusions	Reported. The interpretation distinguishes association and exploratory discrimination from clinically validated prediction and avoids causal claims.
**21**	Discuss generalizability.	Discussion, Limitations	Generalizability is limited by the single-center tertiary-care setting, treated hypertensive population, and small complete-case subgroup.
**Other information**			
**22**	Give the source of funding and the role of funders.	Funding; Conflicts of Interest; Materials and Methods, Section 2.1	Reported. The manuscript states that the study received no external funding and no support or incentives from device manufacturers.

Abbreviations: ABPM, ambulatory blood pressure monitoring; AIx, augmentation index; AIxHR75, augmentation index normalized to 75 beats/min; AoSBP, central aortic systolic blood pressure; BP, blood pressure; cfPWV, carotid–femoral pulse wave velocity; EMM, estimated marginal mean; HT, hypertension without preeclampsia; HT+PE, hypertension complicated by preeclampsia; PE, preeclampsia; PPAmpl, pulse pressure amplification; PWA, pulse wave analysis; ROC, receiver operating characteristic.
